# Development of a rapid and simplified protocol for direct bacterial identification from positive blood cultures by using matrix assisted laser desorption ionization time-of- flight mass spectrometry

**DOI:** 10.1186/s12866-015-0594-2

**Published:** 2015-11-06

**Authors:** Aleksandra Jakovljev, Kåre Bergh

**Affiliations:** Department of Medical Microbiology, St. Olavs Hospital, Trondheim University Hospital, Olav Kyrres gate 17, 7006 Trondheim, Norway; Department of Laboratory Medicine, Children’s and Women’s Health, Faculty of Medicine, Norwegian University of Science and Technology, Trondheim, Norway

**Keywords:** Blood culture, In-house method, MALDI-TOF MS, Bacterial identification, BACTEC FX

## Abstract

**Background:**

Bloodstream infections represent serious conditions carrying a high mortality and morbidity rate. Rapid identification of microorganisms and prompt institution of adequate antimicrobial therapy is of utmost importance for a successful outcome. Aiming at the development of a rapid, simplified and efficient protocol, we developed and compared two in-house preparatory methods for the direct identification of bacteria from positive blood culture flasks (BD BACTEC FX system) by using matrix-assisted laser desorption ionization time-of-flight mass spectrometry (MALDI TOF MS).

Both methods employed saponin and distilled water for erythrocyte lysis. In method A the cellular pellet was overlaid with formic acid on the MALDI TOF target plate for protein extraction, whereas in method B the pellet was exposed to formic acid followed by acetonitrile prior to placing on the target plate.

**Results:**

Best results were obtained by method A. Direct identification was achieved for 81.9 % and 65.8 % (50.3 % and 26.2 % with scores >2.0) of organisms by method A and method B, respectively. Overall concordance with final identification was 100 % to genus and 97.9 % to species level. By applying a lower cut-off score value, the levels of identification obtained by method A and method B increased to 89.3 % and 77.8 % of organisms (81.9 % and 65.8 % identified with scores >1.7), respectively. Using the lowered score criteria, concordance with final results was obtained for 99.3 % of genus and 96.6 % of species identifications.

**Conclusion:**

The reliability of results, rapid performance (approximately 25 min) and applicability of in-house method A have contributed to implementation of this robust and cost-effective method in our laboratory.

## Background

Bloodstream infections are a leading cause of in-hospital mortality and morbidity [1]. Rapid identification of pathogenic microorganisms from blood cultures is crucial for effective antimicrobial therapy [2]. In a large retrospective study of 5,715 patients with septic shock, Kumar et al. found that the survival rate decreased fivefold with inappropriate initial therapy, and it was shown to be the single most powerful risk factor associated with mortality [3].

Processing of bacterial blood cultures is slow due to the incubation time required to reach the detection level and the subsequent subculture onto solid media for final identification. Previous attempts aiming at a more rapid identification of positive blood cultures have included polymerase chain reaction (PCR) and fluorescence in-situ hybridization (FISH) [4–6]. A major shortcoming of these sophisticated techniques is the narrow spectra of organisms detected in a single run. Since the beginning of the twenty-first century, matrix-assisted laser desorption ionization time-of-flight mass spectrometry (MALDI-TOF MS) has been broadly applied in microbiology laboratories for detection of theoretically an unlimited number of bacterial and yeast species [7, 8]. Several methods for direct bacterial identification in positive blood cultures with MALDI –TOF MS have been developed and published during the last years [9–11]. Clinical relevance of rapid identification has been demonstrated with modifications of empirical treatment regimens registered in 13–35 % of patients [12–14]. Disadvantages of previously described in-house procedures are difficulties for implementation in routine laboratory work due to several time-consuming steps and laborious approach. Studies comparing Sepsityper Kit (Bruker Daltonics, Bremen, Germany) with some of the in-house methods have not shown a significant benefit of this commercially available method [15–18].

Taking into consideration both economic factors and hands-on processing time, development of a rapid and easy to implement in-house method became a priority in our laboratory. The aim of this study was to compare the performance of two rapid in-house methods for direct identification of bacteria from positive blood cultures by MALDI-TOF MS and evaluate the applicability for use in routine laboratory work.

## Results

A total of 152 positive blood cultures (149 monomicrobial and three polymicrobial) were prospectively included in the study. Of the 149 monomicrobial blood culture flasks, 82 (55 %) contained Gram-positive organisms and 67 (45 %) contained Gram-negative organisms, determined by Gram stain microscopy and confirmed by definitive bacterial identification with routine methods.

### Successful direct identification from positive blood culture flasks

Direct bacterial identification was achieved for the majority of positive blood cultures. In general, identification of Gram negative organisms was more often successful compared to Gram positive. The level of identification achieved was dependent on the score level used as cut-off. Detailed results obtained for the various microorganisms identified in relation to the score values are presented in Table 1.

**Table 1:** Performance of MALDI-TOF MS identification of 149 monomicrobial blood cultures by in-house method A and in-house method B compared with definitive identification

Definitive identification (with number of isolates)		In-house method A with recommended and reduced cut-off score values								In-house method B with recommended and reduced cut-off score values							
		>2.0	>1.7 < 2.0	<1.7	Concordant id	>1.7	>1.5 < 1.7	<1.5	Concordant id	>2.0	>1.7 < 2.0	<1.7	Concordant id	>1.7	>1.5 < 1.7	<1.5	Concordant id
Gram-positive																	
*Aerococcus urinae*	2	1		1	1	1		1	1			2	0			2	0
*Clostridium hathewayi*	1		1		1	1			1			1	0			1	0
*Enterococcus caseliflavus*	1		1		1	1			1		1		1	1			1
*Enterococcus faecalis*	12	8	3	1	11	11		1	11	1	9	2	10	10	1	1	11
*Enterococcus faecium*	2	1	1		2	2			2		0	2	0		1	1	1
*Enterococcus hirae*	1		1		1	1			1			1	0		1		1
*Lactobacilus rhamnosus* ^*d*^	1			1	0		1		1			1	0			1	0
*Listeria monocytogenes*	1			1	0			1	0			1	0			1	0
*Micrococcus luteus*	1		1		1	1			1		1		1	1			1
*Propionibacterium acnes*	2			2	0			2	0			2.	0			2	0
*Staphylococcus aureus*	17	15	2		17	17			17	7	6	4	13	13	2	2	15
*Staphylococcus capitis*	2	2			2	2			2		1	1	1	1		1	1
*Staphylococcus condimenti*	1		1		1	1			1			1	0		1		1
*Staphylococcus epidermidis*	11	1	6	4	7	7	4		11	1	4	6	5	5	5	1	10
*Staphylococcus hominis*	7	3	1	3	4	4	3		7	3	1	3	4	4	2	1	6
*Streptococcus agalactiae*	4	2	2		4	4			4		4		4	4			4
*Streptococcus anginosus* ^*a*^	1		1		1	1			1			1	0		1		1
*Streptococcus dysgalactiae* ^*f*^	2		2		2	2			2			2	0		2		2
*Streptococcus gordonii*	1			1	0			1	0			1	0			1	0
*Streptococcus mitis* ^*b*^	2		1	1	1	1		1	1	1		1	1	1		1	1
*Streptococcus parasanguinis*	1			1	0		1		1			1	0			1	0
*Streptococcus pneumoniae*	6		1	5	1	1		5	1		1	5	1	1		5	1
*Streptococcus pyogenes*	3	2	1		3	3			3		3		3	3			3
Total	82	35	26	21	61	61	9	12	70	13	31	38	44	44	16	22	60
Total (%)	55%	41.2 %	31.7 %	25.6 %	74.4 %	74.4 %	11 %	14.6 %	85.4 %	15.9 %	37.8 %	46.3 %	53.7 %	53.7 %	19.5 %	26.8 %	73.2 %
Gram-negative																	
*Acinetobacter nosocomialis*	2			2	0		1	1	1			2	0			2	0
*Acinetobacter radioresistens*	1		1		1	1			1	1			1	1			1
*Bacteroides fragilis*	3	2	1		3	3			3		2	1	2	2		1	2
*Capnocytophaga sputigena*	1		1		1	1			1	1			1	1			1
*Citrobacter freundi* ^*e*^	1			1	0		1		0	1			1	1			1
*Citrobacter koseri*	1		1		1	1			1			1	0			1	0
*Enterobacter cloacae complex*	3	1	2		3	3			3	1	2		3	3			3
*Escherichia coli* ^*g*^	35	28	7		35	35			35	18	14	3	32	32		3	32
*Francisella tularensis*	1			1	0			1	0			1	0			1	0
*Haemophilus influenzae*	1		1		1	1			1		1		1	1			1
*Klebsiella pneumoniae*	4	2	2		4	4			4		3	1	3	3	1		4
*Klebsiella oxytoca*	3	2	1		3	3			3	2		1	2	2		1	2
*Moraxella nonliquefaciens*	1			1	0		1		1			1	0			1	0
*Proteus mirabilis*	3	3			3	3			3	1	2		3	3			3
*Providencia rettgeri*	1		1		1	1			1		1		1	1			1
*Pseudomonas aeruginosa*	4	2	1	1	3	3		1	3	1	2	1	3	3	1		4
*Salmonella typhimurium* ^*c*^	1		1		1	1			1		1		1	1			1
*Yersinia enterocolitica*	1		1		1	1			1			1	0			1	0
Total	67	40	21	6	61	61	3	3	63	26	28	14	54	54	2	11	56
Total (%)	45 %	59.7 %	31.3 %	9 %	91 %	91 %	4.8 %	4.8 %	94 %	38.8 %	41.8 %	20.9 %	80.6 %	80.6 %	3 %	16.4 %	83.6 %
Total G + and G - bacteria	149	75	47	7	122	122	12	15	133	39	59	52	98	98	18	33	116
		50.3 %	31.5 %	18.1 %	81.9 %	81.9 %	8.05 %	10 %	89.3 %	26.2 %	39.6 %	34.9 %	65.8 %	65.8 %	12 %	22.1 %	77.8 %

Exclusions from completely achieved concordant identifications

In-house method A: discrepancies on species level results with score values >1.7

^a^ *Streptococcus anginosus* misidentified as *Streptococcus constellatus*

^b^Inability to accurately differentiate between *Streptococcus mitis* and *Streptococcus pneumoniae*

^c^Possibility of *Salmonella spp*. identification only to genus level

Discrepancies on species or genus level results with score values >1.5

^d^ *Lactobacillus rhamnosus* misidentified as *Lactobacillus sharpeae*

^e^ *Citrobacter freundii* misidentified as *Lactobacillus helveticus*

In-house method B: discrepancies on species level results with score values >1.7

^b^Inability to accurately differentiate between *S. mitis* and *S. pneumoniae*

^c^Possibility of *Salmonella spp.* identification only to genus level

Discrepancies on species level results with score values >1.5

^a^ *S. anginosus* misidentified as *S. constellatus*

^f^ *S. dysgalactiae* misidentified as *S. pyogenes*

^g^One isolate of *Escherichia coli* was identified with score 1.7 and classified in table as >1.7 < 2.0

Identification of microorganisms was achieved more frequently by in-house method A in comparison to in-house method B (p < 0.001). In-house method A identified 75/149 (50.3 %) to species level by using a score >2.0 and 47/149 (31.5 %) to genus level at a score >1.7 < 2.0, whereas in-house method B identified 39/149 (26.2 %) to species and 59/149 (39.6 %) to genus level. Direct correct identification was achieved for 122/149 (81.9 %) of positive blood cultures by in-house method A, including 91 % of Gram negative and 74.4 % of Gram positive bacteria. Also by applying reduced cut-off score of >1.7 for species identification and >1.5 < 1.7 for genus, in-house method A was superior to in-house method B (122/149 vs 98/149 at species level and 12/149 vs 18/149 at genus level). Direct correct identification from blood cultures was achieved for 133/149 (89.3 %) at a cut-off >1.5 by using in-house method A.

### Discrepancies

The concordance of direct identification and definitive identification was very high: 100 % - 99.3 % to genus level and 97.9 %–96.6 % to species level at the recommended and reduced cut-off, respectively. Using the less stringent cut-off criteria, only one potential misidentification at the genus level was observed: one *Citrobacter freundii* isolate by direct identification using in-house method A yielded a score of 1.513 as *Lactobacillus helveticus* closely followed by three next suggestions of *C. freundii* with scores 1.474–1.453-1.420. Otherwise, only five minor discrepancies were observed: 1) one *Streptococcus mitis* isolate was identified as *Streptococcus pneumoniae* directly by both preparatory methods; 2) one *Streptococcus anginosus* isolate was identified as *Streptococcus constellatus* (in-house method A score 1.745, in-house method B score 1.694); 3) one *Salmonella typhimurium* yielded scores of 1.890 and 1.710 as *Salmonella spp.*; 4) one *Streptococcus dysgalactiae* isolate yielded successively ranking identifications as *Streptococcus pyogenes* (score 1.628), *S. dysgalactiae* (scores 1.589 and 1.51) by in-house method B; and 5) *Lactobacillus rhamnosus* by in-house method A yielded a score of 1.568 as *Lactobacillus sharpeae.*

### Organism-specific issues

Classification of *Enterobacter cloacae complex* as final identification to species level was accepted considering similar molecular pattern of six *Enterobacter spp*. assigned to this complex. Various *Enterobacter* species identifications with score values >2.0 were obtained with MALDI-TOF MS directly from blood cultures and from agar growth. Only one out of six *S. pneumoniae* isolates was reliably identified directly (score 1.961 and 1.757 by the two preparatory methods). In four of the five positive blood cultures with negative direct identification, signs of cell autolysis were evident by Gram stain microscopy from blood culture broth.

### Polymicrobial blood cultures

Three patients 3/152 (2 %) were detected with polymicrobial blood cultures, where only one species was successfully identified direct by MALDI-TOF MS (Table 2).

**Table 2:** Definitive identification and direct MALDI-TOF MS identification of three polymicrobial blood cultures

No. of positive blood culture flasks /No. of collected blood culture flasks	Definitive identification of mixed culture	Direct identification from blood cultures by MALDI-TOF MS using in-house method A and B (score values)
4 / 4 4 / 4	*Staphylococcus epidermidis* *Enterococcus faecalis*	*Staphylococcus epidermidis* (1.765 and <1.7)
6 / 6 6 / 6 2 / 6	*Escherichia coli* *Streptococcus mitis* *Streptococcus oralis*	*Escherichia coli* (2.139 and 2.091)
1 / 6 1 / 6	*Staphylococcus hominis* *Staphylococcus epidermidis*	*Staphylococcus hominis* (1.838 and 1.836)

## Discussion

In this study we have assessed the diagnostic value and applicability for routine laboratory use of two rapid in-house methods. Performance of in-house method A produced significantly better results compared to in-house method B (*p* < 0.001). In-house method A, provided the higher level of identification, detecting accurately 81.9 % of organisms from positive blood cultures with no discordance at genus level and only three minor discrepancies at species level using the manufacturer’s cut-off criteria.

Excellent reliability and applicability of in-house method A have contributed to its implementation in our routine laboratory work. The method is easy to use and rapid, performed on a small volume sample (1 ml) and using saponin for lysis of erythrocytes. Different methods have been employed by several authors for lysis of erythrocytes, including saponin [9, 16, 17], followed by time-consuming extraction procedures including additional acetonitrile and ethanol steps as described in the Sepsityper Kit and some other in-house methods [9–11, 15–21]. The advantages of in-house method A are its rapidness and simplicity obtained by adding of only formic acid for cell wall disruption and extraction [22]. The identification results can be obtained in approximately 25 min after registration of a positive signal in BACTEC blood culture system. Additional practical points favouring our in-house method are its robustness, cost-effectiveness and low economic burden. Currently in our laboratory positive blood cultures are processed for MALDI-TOF analysis once a day during the morning hours. As we are in the transition to extend laboratory opening hours to 10 pm, we intend to process positive blood cultures at least twice daily.

Considering in-house method B some practical disadvantages were noticed. Due to its potential for refinement, some issues are mentioned here since we are unaware of previous presentations of rapid direct identification by using the mixture of pellet, formic acid and acetonitrile. Combination of a formic acid and acetonitrile step was expected to provide better protein extraction, but instability of the method was observed on several occasions, possibly caused by strong time dependence during the process. We have observed that prolongation of reaction time with acetonitrile for only a few minutes did influence the identification providing inconsistent results. In routine laboratory work, where multiple positive blood cultures are to be tested successively, it would be difficult to adapt work-flow to this time dependence factor. Some other excluding points were the necessity for absolutely gentle mixing of pellet during extraction with acetonitrile, and in some cases adjustment of volumes of formic acid and acetonitrile added dependent on variable sizes of pellet. It is possible that those disturbing factors can be reduced by combination of formic acid and acetonitrile directly on MALDI TOF plate, as recently described by Barnini et al. [23].

Previously, considerable improvement of results obtained by direct identification from blood cultures has been observed by modification of threshold values [19–21, 24, 25]. The results from our study corroborate the advantage of lowering the cut-off scores applied. By adjustment of cut-off score values for species and genus level identification to >1.7 and >1.5 < 1.7, respectively, 89.3 % of organisms were identified by method A, including 94 % of Gram negative and 85.4 % of Gram positive bacteria. The benefit of reduced cut-off score values was particularly evident for higher confident species identification of 81.9 % of organisms, comprising 91 % of Gram negative and 74.4 % of Gram positive bacteria. Genus discordance was observed only in one isolate of *Citrobacter freundii* misidentified directly as *Lactobacillus helveticus* with a score of 1.513 close to the lower limit. Additionally five minor discrepancies at species level were observed, most often of little significance, as limitation of MALDI-TOF MS in differentiating within the *Streptococcus mitis* group and serotypes of *Salmonella enterica* is well known. Overall concordance with definitive identification was 99.3 % to genus and 96.6 % to species level with cut-off score values >1.5.

Identification problems regarding lower score values (<1.4) were observed in the cases of some of the viridans streptococci, *Corynebacterium spp*. and *Propionibacterium acnes*. Insufficient concentration of bacteria in the blood culture flasks, as well as insufficient proteomic profiles in database, could theoretically account for not reliable identifications [11, 19, 26, 27]. A minimum concentration of 10^6^ CFU/mL was considered necessary for obtaining high quality spectra [11, 26]. However, some authors report a higher sensitivity of MALDI-TOF MS with sufficient signal-to-noise ratio generated from a sample containing less than 10^4^ microorganisms/mL [8, 28, 29].

The majority of *S. pneumoniae* isolates (5/6) could not be identified directly with reliable score values, yielding scores at the level of negative blood cultures (1.1–1.2). We hypothesize that insufficient proteomic material, possibly due to autolysis, could be an explanation. Similar proportion of negative results was reported by Prod’hom G et al. [30], where 8/10 isolates of *S. pneumoniae* did not achieve reliable scores.

The majority of directly identified isolates of *Enterobacteriaceae, Staphylococcus aureus* and coagulase negative staphylococci (CNS), *Enterococcus spp.,* non-fermentative Gram negative bacteria as well as anaerobic bacteria, provided reliable identification results.

In our study, polymicrobial blood cultures were detected in 3/152 (2 %), which is a lower rate compared to other publications, describing frequencies from 5.5–9 % [26, 31]. In each of three cases, only one of the organisms was correctly identified by direct MALDI-TOF MS.

A beneficial impact of rapid microbial identification from positive blood cultures on adjustment of empirical antimicrobial therapy has been shown in previous studies [12–14]. Systematic evaluation of modifications of antibiotic therapy was beyond the scope of the present study. Nevertheless, several cases of therapeutic adjustments could be considered reasonable based on intrinsic antimicrobial resistance or known local resistance patterns of identified bacterial species [32]. Examples of the latter are cases of *E. faecalis* or *E. faecium* bacteremia when preemptive therapy was a cephalosporin, or identification of *Acinetobacter spp*. and some of the *Enterobacteriaceae* to the genus level, that could indicate a need for switching to broader therapy from cephalosporin to a carbapenem. Also, rapid differentiation between *S. aureus* and CNS could be helpful both for the choice of therapy (cloxacillin or vancomycin) and initiation of search for a potential focus of bloodstream infection.

When rare pathogenic organisms were identified directly from blood cultures, the usefulness of rapid identification was demonstrated. In our study, identification of *Listeria monocytogenes* from blood culture was achieved as not reliable with a best score of 1.497. Nevertheless, the various *Listeria* species, including *L. monocytogenes*, ranking successively as first seven proposals on identifications list, provided valuable information of possible listeriosis prompting immediate performance of *L. monocytogenes* PCR which was positive. According to observations by Moussaoui et al. [19] when at least four successive species proposals with first scores >1.4 were obtained directly from blood cultures, species identification was never false in their series. As a special case, we had one isolate of *Y. enterocolitica* identified directly from blood culture flask with score value 1.733, with four successive proposals of *Y. enterocolitica* followed by six various *Yersinia spp.* In the case of *Francisella tularensis* isolate, the suspicion of this possible pathogen was raised after acridine orange microscopy, performed routinely for all negative Gram microscopy. Positive acridine orange stain and knowledge of endemic tularaemia infections in Norway [33, 34], led to request of direct *F. tularensis* PCR with positive result obtained during the next hours. After diagnosing tularaemia, we have tried to identify *F. tularensis* directly by MALDI-TOF MS. Unfortunately, Bruker Taxonomy database used in routine laboratory diagnostic does not include *F. tularensis* and SR Taxonomy database (Special Risk bioterror agents) was not accessible at the time of testing.

## Conclusion

To summarize, in this evaluation study we have successfully shown that a rapid in-house method with simple formic acid extraction step provides reliable identification results. At the same time, it is applicable at a low cost and easy to implement in routine laboratory work. Most importantly, the rapid identification of bacterial isolates from blood cultures enables improved patient care, when the antibiotic therapy started is considered inadequate, knowing species or genus identification prior to obtaining results of susceptibility analysis. For further improvement of patient therapy in our hospital, it would be necessary to include antimicrobial susceptibility testing directly from positive blood cultures using an automated system [35].

## Materials and methods

### Blood cultures

A total of 152 positive blood cultures (collected from 146 patients) were prospectively included in the period from May to September 2014. Using aseptic procedures, blood was drawn and inoculated into aerobic and anaerobic blood agar flasks, and for children where only small volume (<3 ml) was obtainable, into pediatric blood culture flasks (BACTEC Plus/F Aerobic, BACTEC Plus/F Anaerobic and BACTEC Peds Plus/F, Becton Dickinson, BD Diagnostics, USA). Incubation was performed in automated blood culture system BD BACTEC FX. Positive blood cultures were analysed with direct MALDI-TOF MS identification immediately after reporting signal between 8 am to 5 pm, or next morning, if becoming positive after 5 pm. Incubation time after achievement of growth detection level (positive signal) was thus prolonged for the flasks analysed next day. Microscopy of Gram stained smears was performed for all positive blood culture flasks and in the cases of monomicrobial bacterial morphology, the first flask from each patient was included for direct MALDI-TOF MS analysis. Three patients from whom two different organisms were isolated from separate blood culture flasks taken approximately at the same collection time, were analysed as monomicrobial blood cultures. Only three patients (3/152) were detected with true polymicrobial blood cultures. All positive blood cultures were subcultured on agar media according to Gram stain results: blood agar, chocolate agar, MacConkey agar and special agars when required (Francisella agar, BD Diagnostics, Difco and Listeria Selective agar, Oxoid, antibiotics from MAST group) at 37 °C in an atmosphere with 5 % CO_2_ for 24–48 h (Francisella agar without CO_2_)_._ Positive anaerobic blood cultures were additionally subcultured on Fastidious Anaerobic agar (Oxoid) and incubated in an anaerobic chamber for 48 h or longer if required. The results of direct identification from blood culture flasks were compared with definitive identification obtained from bacterial growth on solid agar media and identified by routine methods with high confidence level, including rapid and standard biochemical tests (Slidex Pneumo-Kit BioMeriéux, Optochin disc BD BBL™, Pastorex™ Strep Bio-Rad, Monostaph plus agglutination test for rapid identification of *Staphylococcus aureus*, Bionor Laboratory AS Norway, Api 20 STREP BioMeriéux), MALDI-TOF MS and, in selected instances, in-house PCR for *Francisella tularensis* and *Listeria monocytogenes.* The present study was performed on bacterial isolates and did not take into account identifiable patient data. The study did not influence on preliminary treatment decisions as processing of blood cultures was performed in routine manner according hospital procedures. Thus, patient consent is not required as the study can be assessed as quality assurance which the laboratory is expected to perform.

## Methods

### In-house-method A

The first in-house method is a modification of the method described by Martiny et al. [15] by adding an additional formic acid extraction step previously successfully applied for direct organism extraction on MALDI TOF stainless target plate [36–38]. Briefly, 1 mL of positive blood culture broth was collected in 1.5 mL capped Eppendorf tube and 200 μL of 5 % saponin lysis buffer used for lysis of erythrocytes (SERVA Electrophoresis GmbH, Heidelberg, product nr: 34655.02) was added. After short vortexing the tube was left for 10 min at room temperature and then centrifuged for 2 min at 13,200 rpm in a MiniSpin centrifuge (Eppendorf, Germany) and the supernatant was carefully removed. The pellet with residuals of lysed erythrocytes was washed with 1 mL of sterile distilled water, with repeating of the centrifugation and supernatant removal step. The cell pellet was smeared on a MALDI-TOF MS target plate, overlaid with 1 μL of 70 % formic acid and gently mixed. When dry, the deposit was covered with 0.8 μL of matrix solution (10 mg/mL α-cyano-4-hydroxycinnamic acid [HCAA] in 50 % acetonitrile-2.5 % trifluoroacetic acid) (Bruker Daltonics, GmbH, Germany).

### In-house method B

The lysing process was identical to that described above, but the method was modified in the extraction step by a combination of formic acid for cell wall disruption and acetonitrile for protein extraction [10, 22]. Briefly, after centrifugation and supernatant removal step, 20 μL of formic acid was added to the pellet and gently mixed using a micropipette tip, avoiding aspiration or use of vortex. After 5 min at room temperature, 20 μL of acetonitrile was added, mixed and allowed to rest for 5 min. 1 μL of mixture was placed on a target plate and after air drying, covered by matrix.

### MALDI-TOF MS analysis

The samples were analysed using MALDI-TOF MS on a Microflex LT mass spectrometer (Bruker Daltonics) with Biotyper 3.2 software database (comprising 4,613 spectra). Calibration of MALDI TOF instrument was performed daily with Bruker Daltonics GmbH positive control strain *Escherichia coli* DH5α.

### Statistical analysis

For comparison of sensitivity McNemar’s test was used. Statistical significance was considered at *p* < 0.05.
